# Increased Mortality from Lung Cancer and Bronchiectasis in Young Adults after Exposure to Arsenic *in Utero* and in Early Childhood

**DOI:** 10.1289/ehp.8832

**Published:** 2006-03-27

**Authors:** Allan H. Smith, Guillermo Marshall, Yan Yuan, Catterina Ferreccio, Jane Liaw, Ondine von Ehrenstein, Craig Steinmaus, Michael N. Bates, Steve Selvin

**Affiliations:** 1 Arsenic Health Effects Research Program, University of California, Berkeley, California, USA; 2 Pontificia Universidad Católica de Chile, Santiago, Chile; 3 Office of Environmental Health Hazard Assessment, California Environmental Protection Agency, Oakland, California, USA; 4 School of Public Health, University of California, Berkeley, California, USA

**Keywords:** arsenic, bronchiectasis, childhood exposure, chronic obstructive pulmonary disease, drinking water, *in utero* exposure

## Abstract

Arsenic in drinking water is an established cause of lung cancer, and preliminary evidence suggests that ingested arsenic may also cause nonmalignant lung disease. Antofagasta is the second largest city in Chile and had a distinct period of very high arsenic exposure that began in 1958 and lasted until 1971, when an arsenic removal plant was installed. This unique exposure scenario provides a rare opportunity to investigate the long-term mortality impact of early-life arsenic exposure. In this study, we compared mortality rates in Antofagasta in the period 1989–2000 with those of the rest of Chile, focusing on subjects who were born during or just before the peak exposure period and who were 30–49 years of age at the time of death. For the birth cohort born just before the high-exposure period (1950–1957) and exposed in early childhood, the standardized mortality ratio (SMR) for lung cancer was 7.0 [95% confidence interval (CI), 5.4–8.9; *p* < 0.001] and the SMR for bronchiectasis was 12.4 (95% CI, 3.3–31.7; *p* < 0.001). For those born during the high-exposure period (1958–1970) with probable exposure *in utero* and early childhood, the corresponding SMRs were 6.1 (95% CI, 3.5–9.9; *p* < 0.001) for lung cancer and 46.2 (95% CI, 21.1–87.7; *p* < 0.001) for bronchiectasis. These findings suggest that exposure to arsenic in drinking water during early childhood or *in utero* has pronounced pulmonary effects, greatly increasing subsequent mortality in young adults from both malignant and nonmalignant lung disease.

The International Agency for Research on Cancer (IARC) has classified arsenic in drinking water as a group 1 carcinogen that causes skin cancer, bladder cancer, and lung cancer (IARC 2002). Substantial evidence supports the biologic plausibility that exposure to arsenic can lead to skin and bladder cancer. For example, arsenic concentrates in the skin and is known to cause nonmalignant skin lesions [National Research Council (NRC) 2001], and the major pathway of excretion is in urine, giving plausibility to increased bladder cancer rates (NRC 2001). Although it is known that inhalation of arsenic may cause lung cancer, the findings of increased lung cancer mortality after ingestion in drinking water were unexpected because all other known lung carcinogens act via inhalation. However, the evidence based on multiple studies in Taiwan (Chen and Wang 1990; Chen et al. 1985, 1988; Wu et al. 1989), Chile (Ferreccio et al. 2000; Smith et al. 1998), Argentina (Hopenhayn-Rich et al. 1998), and Japan (Tsuda et al. 1989, 1995) is sufficient to conclude that there is a causal relationship. In fact, lung cancer is the main long-term cause of death from ingesting arsenic in drinking water (NRC 2001). In region II of Chile, which includes the city of Antofagasta, overall lung cancer mortality rates for men and women were previously found to be at least 3-fold higher than for the rest of Chile (Smith et al. 1998), and lung cancer relative risk estimates increased nearly 9-fold in those with the highest exposures (Ferreccio et al. 2000).

Several known lung carcinogens cause chronic nonmalignant respiratory diseases, including cigarette smoking, which causes chronic obstructive pulmonary disease (COPD); asbestos, which causes asbestosis; and silica, which causes silicosis. To date, however, relatively little attention has been given to whether or not ingestion of arsenic in drinking water causes nonmalignant pulmonary disease. The first reports of chronic respiratory symptoms came from small investigations in Antofagasta in the 1970s (Zaldivar 1974, 1977, 1980; Zaldivar and Ghai 1980). Before 1958, the water supply in the main city of Antofagasta had an arsenic concentration of about 90 μg/L. A growing population led to supplementation of Antofagasta’s water supply in the late 1950s with water from rivers with arsenic concentrations near 1,000 μg/L. Because this area is among the driest places on Earth, there are very few individual water supplies, and almost everyone drinks water from the same municipal sources. After the installation of a new treatment plant in 1971, arsenic levels in Antofagasta water dropped abruptly to about 90 μg/L and have been progressively reduced further in recent years (Ferreccio et al. 2000). These data are shown in Figure 1.

In a 1998 publication concerning region II, increased COPD mortality was reported for the 30- to 39-year age group (Smith et al. 1998). Based on the time period in which mortality was assessed (1989–1993), subjects in the 30- to 39-year age group would have been *in utero* or young children at the time of the peak exposure period in Antofagasta. These results were based on a small number of cases but were later supported by findings from other arsenic-exposed regions. For example, increases in symptoms of chronic respiratory disease were found to be associated with arsenic ingestion in studies in West Bengal, India (De et al. 2004; Guha Mazumder et al. 2000) and Bangladesh (Milton and Rahman 2002). Recently, two studies in West Bengal involving participants with arsenic-caused skin lesions reported major deficits in pulmonary function (von Ehrenstein et al. 2005) and a 10-fold increase in prevalence of bronchiectasis identified by high-resolution computed tomography (Guha Mazumder et al. 2005).

The distinct period of high arsenic exposure in Antofagasta from 1958 through 1970 offers the opportunity to investigate the health effects of early-life arsenic exposure. In this study, we take advantage of this unique situation in order to assess adult mortality in those born during the high-exposure period who would have experienced exposure *in utero* as well as early childhood, and those born just before 1958 who would have experienced high exposure during childhood but not *in utero*.

## Materials and Methods

We obtained computerized mortality data for 1989–2000 from the Ministry of Health for all 13 regions of Chile. Deaths were divided into two groups: those who were residents of Antofagasta and neighboring Mejillones, cities that have the same water source; and those who were residents in all regions of Chile other than region II, in which Antofagasta and Mejillones are located. Two birth cohorts were defined for this investigation: those born in the period 1958–1970 (probable *in utero* exposure if resident in Antofagasta/Mejillones) and those born in 1950–1957 (probable childhood exposure if born in Antofagasta/Mejillones). Causes of death were coded according to the *International Classification of Diseases, 9th Revision* (ICD-9; World Health Organization 1978), including lung cancer (ICD-9 code 162) and chronic respiratory disease (ICD-9 codes 490, 491, 492, 494, and 496). We obtained annual estimates of the population living in Antofagasta/Mejillones in region II, and for the rest of Chile excluding region II, for 1989–2000 from the National Institute of Statistics (Instituto Nacional de Estadísticas) stratified by age and sex.

In 2000, the most recent year for which mortality data are available, the oldest persons in the first birth cohort born in the period 1950–1957 would have been 50 years old. We therefore calculated standardized mortality ratios (SMRs) for men and women separately, 30–49 years of age, using 10-year age groups (30–39 and 40–49 years) for standardization. Mortality in younger ages was not included because death from lung cancer or chronic respiratory disease is extremely rare in individuals < 30 years of age. We calculated SMRs as the observed number of deaths divided by the expected number of deaths, using all regions in Chile outside of region II as the referent population. We estimated SMRs for lung cancer, for bronchiectasis, and for other COPD causes of death excluding bronchiectasis, and also for all other causes of death excluding lung cancer and COPD. We calculated tests of significance and confidence intervals (95% CIs) based on the Poisson distribution (Selvin 1995). In view of the clear direction of the *a priori* hypotheses for arsenic and both malignant and nonmalignant pulmonary diseases, we conducted one-tailed tests of significance for increases in these outcomes. We assessed tests for effect modification by age group (comparing 30–39 and 40–49 year age groups) and tests for effect modification by sex and for differences between those born in 1950–1957 and 1958–1970 by testing the pertinent Poisson regression interaction terms with two-tailed tests.

## Results

SMRs for lung cancer and COPD are given in Table 1 for the 30–39 and 40–49 age groups separately and combined and for men and women separately and combined. Based on the Poisson regression interaction terms, there was no evidence of differences in rate ratios between 30–39 and 40–49 age groups for lung cancer and COPD causes of death, so we focused on the SMRs for the overall age range 30–49 years. For lung cancer, the SMR for 30–49 years of age was increased for those born in the period 1950–1957 for both men (SMR = 8.2; 95% CI, 6.2–10.8, *p* < 0.001) and women (SMR = 4.7; 95% CI, 2.7–7.7; *p* < 0.001). The lung cancer SMR was also increased for those born in 1958–1970 (women: SMR = 2.9; 95% CI, 0.6–8.5; *p* = 0.087; men: SMR = 8.1; 95% CI, 4.3–13.9; *p* < 0.001). Concerning COPD mortality, bronchiectasis SMRs were markedly increased for both men and women, especially for those born in the high-exposure period 1958–1970 (women: SMR = 50.1; 95% CI, 20.0–103; *p* < 0.001; men: SMR = 36.4; 95% CI, 4.1–132; *p* = 0.001). SMRs for other COPD causes of death excluding bronchiectasis were elevated, but much less than for bronchiectasis. Finally, for all other causes of death combined, there was little evidence of increased mortality for either birth cohort, as shown in Table 1.

**Figure 2:**
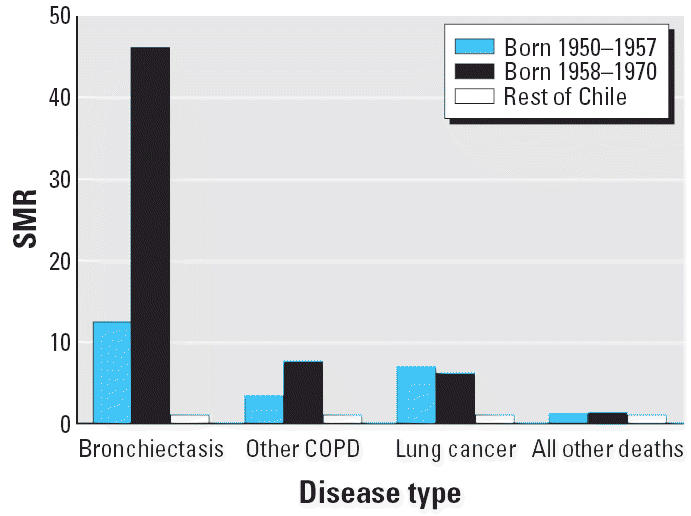
COPD SMRs for Antofagasta/Mejillones for individuals 30–49 years of age, pooled.

The lung cancer relative risks are higher for men than for women, but the CIs for women are wide because of the relatively small numbers and overlap the lung cancer SMR for men (point estimate for men 30–49 years of age, 8.1; 95% CI for women, 0.6–8.5; Table 1). Testing Poisson regression interaction terms, there was little evidence of effect modification by sex for the period 1950–1957 (*p* = 0.23), but testing for effect modification for those born in the period 1958–1970 yields a *p*-value of 0.04, with higher relative risks for men than for women (8.1 for men and 2.9 for women). The pooled results are presented in Table 1 and Figure 2. They show that lung cancer rates are greatly increased for both those born in 1950–1957 with childhood exposure and for those born in 1958–1970 who would have experienced *in utero* exposure. However, for bronchiectasis, and to a lesser extent for other COPD mortality, the SMRs are much higher for those born in 1958–1970 (SMR = 46.2; 95% CI, 21.1–87.7; *p* < 0.001) than for those born in 1950–1957 before the very high exposures started (SMR = 12.4; 95% CI, 3.3–31.7; *p* < 0.001; Poisson regression test for difference in bronchiectasis rate ratios for the two periods, *p* = 0.02).

## Discussion

Region II of Chile provides a unique opportunity to investigate arsenic health effects. It is one of the driest areas of the world, and water used in major cities and towns comes from single sources with known arsenic concentration. Furthermore, there was an abrupt onset of high exposure in 1958 in Antofagasta, the major city of region II with a population at that time of about 200,000 (Zaldivar 1974), and an abrupt reduction in exposure in 1971 when the first large arsenic removal plant in the world was installed there. Such clear-cut exposure patterns are rare in environmental epidemiology, except perhaps radiation exposure from use of the atomic bomb in Hiroshima and Nagasaki and, to a lesser extent, ionizing radiation from accidents at nuclear reactors.

The magnitude of the effects found on lung cancer and bronchiectasis mortality has no parallel with effects of other environmental exposures occurring *in utero* and/or in early childhood. No lung cancer cases were reported in 40 years among the *in utero*–exposed survivors of the atomic bombing of Hiroshima and Nagasaki (Yoshimoto et al. 1988). Children with the highest gamma radiation exposure in Hiroshima and Nagasaki < 10 years of age did not experience increased lung cancer risks as adults, but those exposed in the age range of 10–19 years had lung cancer relative risk estimates of about 2.5 those of young adults 30–39 years of age (Shimizu et al. 1990, figure 2). The evidence for an effect of childhood exposure to environmental tobacco smoke on adult lung cancer rates is mixed, with a meta-analysis finding no overall evidence of increased risks (Boffetta et al. 2000). However, a prospective study reported a relative risk estimate of 3.6 (95% CI, 1.2–11.1) based on four lung cancer cases among those with “many hours” of daily exposure (Vineis et al. 2005). By contrast, we report here a total of 84 deaths from lung cancer after childhood exposure to high concentrations of arsenic in drinking water in Chile, a 6- and 7-fold increase above rates in the rest of Chile (Table 1).

Some supportive evidence provides biologic plausibility for arsenic having effects *in utero*. Arsenic crosses the placenta in animals and humans, and there is human evidence that arsenic is a developmental toxicant affecting birth weight and reproductive outcomes (Concha et al. 1998; Hanlon and Ferm 1987; Hopenhayn et al. 2003; Hopenhayn-Rich et al. 1999, 2000). A study conducted in Bangladesh showed an increased risk for stillbirth [odds ratio (OR) = 2.5; 95% CI, 1.5–4.9] and spontaneous abortion (OR = 2.5; 95% CI, 1.5–4.3) in women with current arsenic exposure ≥ 100 μg/L in water (Milton et al. 2005), and a study in West Bengal found increased risks of stillbirths (OR = 6.1; 95% CI, 1.5–24.0) (von Ehrenstein et al. 2006). As a whole, these epidemiologic data provide evidence that arsenic exposure *in utero* could be associated with a number of adverse effects. The present study, however, is the first to provide evidence that early-life exposures may produce effects manifesting in adults.

Oral-dose animal studies demonstrate arsenic teratogenicity (Chattopadhyay et al. 2002; Vahter 1994). Of particular relevance to our study is evidence that arsenic is a transplacental carcinogen in mice (Waalkes et al. 2000). Female offspring of pregnant mice that were given high doses of arsenic in their drinking water developed tumors at multiple sites, including the lung, with lung carcinoma increased to 5 of 24 (21%) compared with 0 of 25 (0%) in the unexposed controls.

Strengths of our study include the extensive documentation of arsenic in drinking water in the Antofagasta water system. Records of arsenic levels in Antofagasta have been kept for the last 50 years, and almost all residents drink from the same water supply. One potential limitation of this study is that it is ecologic in nature, because overall mortality rates in the cities of Antofagasta/Mejillones were compared with those of the rest of Chile. Residence was determined from death certificates and relates to residence at the time at death. We cannot be certain that those manifesting the increased mortality were actually born in Antofagasta/Mejillones. However, the increases in relative risks are far too great to result from bias due to in-migration of very high-risk persons born elsewhere. We conclude that the effects are most probably due to arsenic in the water and that, if anything, they are diluted by in-migration of people who were born and grew up elsewhere in Chile.

The study’s weakness lies in its reliance on death certificates, even though Chilean mortality records are well documented: Laws require that deaths be registered with the Civilian Registration Service (Servicio de Registro Civil), whereas another branch of government, the National Institute of Statistics (Instituto Nacional de Estadísticas), oversees validation of the generated data. Death certificates are coded according to the standard ICD, and the 1996 World Health Statistics cited Chile as having 100, 100, and 98% of all estimated deaths registered for the years 1991, 1993, and 1994, respectively (World Health Organization 1998). However, although death certificates provide reasonably good data for lung cancer studies, they have known limitations for identifying death from chronic respiratory disease (Selikoff and Seidman 1992). This leads one to question whether medical practices in region II might have led to overdiagnosis of chronic respiratory disease as a cause of death placed on death certificates, particularly deaths from bronchiectasis. However, separating out the findings concerning bronchiectasis from other COPD causes of death was conducted with a clear *a priori* hypothesis. Although previous mention had been made in the literature of bronchiectasis and arsenic, it was the recent finding of a 10-fold increase in bronchiectasis prevalence in persons with high exposure to arsenic and arsenic-caused skin lesions in West Bengal, India (Guha Mazumder et al. 2005), that led us specifically to evaluate bronchiectasis in this study.

Although smoking is strongly associated with mortality from lung cancer and COPD, confounding due to smoking is unlikely. Smoking is not a strong risk factor for bronchiectasis and so would not confound our findings regarding this disease (Barker 2002). And even in extreme form, confounding could not produce the marked elevation of lung cancer relative risks we have found (Axelson 1980). In addition, smoking data do not indicate higher smoking rates in region II than in the rest of Chile, according to a national survey conducted in 1990 (Ministerio de Planificación y Cooperación Nacional Republica de Chile 1992). The survey included the two largest cities in region II (Antofagasta and Calama), which constitute 80% of the region II population; the proportion of smokers in these two cities was found to be lower than the rest of Chile, and the two cities also had a smaller proportion of people who smoked more than one pack per day (Table 2) (Smith et al. 1998). Although there is some evidence that exposure of children to passive smoking in their homes increases the risk of adult lung cancer (Lee et al. 2000; Vineis et al. 2005), an earlier meta-analysis estimated the relative risk to be 0.91 (95% CI, 0.8–1.05) (Boffetta et al. 2000). Even if passive smoking does increase the risk of adult lung cancer, such exposure occurs throughout Chile. Finally, occupational exposures to arsenic, such as in the mining and refining of copper, could contribute to COPD and lung cancer mortality, but these occupational exposures mainly involve men, and our study found similar increases in mortality in both men and women.

In conclusion, we have demonstrated pronounced increases in mortality from lung cancer and bronchiectasis in persons with probable exposure to high concentrations of arsenic in drinking water *in utero* and early childhood. These findings are important in that they provide some of the first human evidence of effects from environmental exposures to toxic chemicals *in utero* and early childhood resulting in disease in adults. A marked increase in mortality in young adults is also of public health importance and should be taken into consideration in setting arsenic drinking water standards.

## Figures and Tables

**Figure 1:**
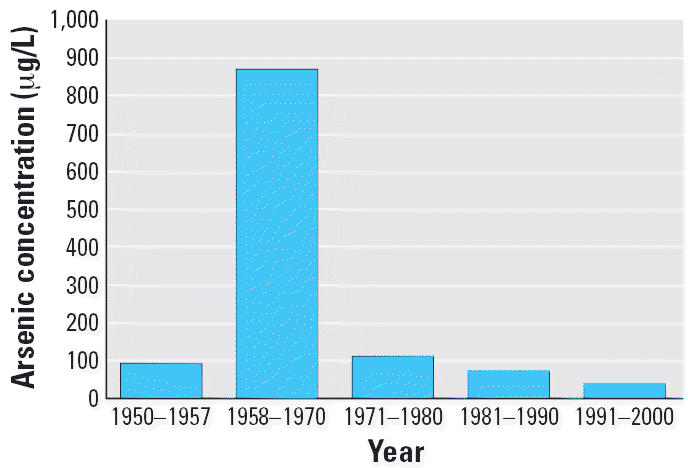
Arsenic concentrations in Antofagasta/Mejillones water by year. An arsenic removal plant was installed in 1971.

**Table 1:** SMRs for bronchiectasis, other COPD, all other deaths, and lung cancer for Antofagasta/Mejillones, for ages 30–49, for men and women both separately and pooled.

			Born 1950–1957				Born 1958–1970			
Age (years)	Sex	Cause of death	O	E	SMR (95% CI)	*p*-Value	O	E	SMR (95% CI)	*p*-Value
30–39	Male	Lung cancer	15	1.17	12.8 (7.1–21.1)	< 0.001	12	1.30	9.2 (4.8–16.1)	< 0.001
		Bronchiectasis	3	0.15	19.4 (4.0–56.8)	0.001	2	0.05	36.4 (4.1–132)	0.001
		Other COPD	1	0.21	4.7 (0.1–26.0)	0.193	1	0.46	2.2 (0.1–12.1)	0.368
		All other deaths	129	155.78	0.8 (0.7–1.0)	0.987	305	304.41	1.0 (0.9–1.1)	0.494
30–39	Female	Lung cancer	2	0.48	4.2 (0.5–15.1)	0.084	3	0.83	3.6 (0.7–10.5)	0.052
		Bronchiectasis	0	0.04	0	—	6	0.14	42.9 (15.7–93.4)	< 0.001
		Other COPD	2	0.14	13.9 (1.7–50.2)	0.009	4	0.33	12.2 (3.3–31.2)	< 0.001
		All other deaths	74	64.95	1.1 (0.9–1.4)	0.145	145	113.73	1.3 (1.1–1.5)	0.003
30–39	Pooled	Lung cancer	17	1.65	10.3 (6.0–16.5)	< 0.001	15	2.14	7.0 (3.9–11.6)	< 0.001
		Bronchiectasis	3	0.19	15.8 (3.2–46.0)	0.001	8	0.19	41.1 (17.7–80.9)	< 0.001
		Other COPD	3	0.36	8.4 (1.7–24.5)	0.006	5	0.79	6.3 (2.1–14.8)	0.001
		All other deaths	203	220.73	0.9 (0.8–1.1)	0.891	450	418.14	1.1 (1.0–1.2)	0.064
40–49	Male	Lung cancer	37	5.14	7.2 (5.1–9.9)	< 0.001	1	0.29	3.4 (0.01–18.9)	0.255
		Bronchiectasis	0	0.10	0	—	0	0	0	—
		Other COPD	3	1.30	2.3 (0.5–6.7)	0.144	1	0.10	10.2 (0.3–56.8)	0.093
		All other deaths	270	292.37	0.9 (0.8–1.0)	0.911	21	19.66	1.1 (0.7–1.6)	0.411
40–49	Female	Lung cancer	14	2.90	4.8 (2.6–8.1)	< 0.001	0	0.20	0.0	—
		Bronchiectasis	1	0.04	27.6 (0.7–154)	0.036	1	0.0	0.0	—
		Other COPD	2	0.76	2.6 (0.3–9.5)	0.177	1	0.04	27.4 (0.7–153)	0.036
		All other deaths	178	147.78	1.2 (1.0–1.4)	0.009	17	11.92	1.4 (0.8–2.3)	0.097
40–49	Pooled	Lung cancer	51	8.04	6.3 (4.7–8.3)	< 0.001	1	0.50	2.0 (0.01–11.2)	0.391
		Bronchiectasis	1	0.13	7.5 (0.2–42.0)	0.124	1	0.0	0.0	—
		Other COPD	5	2.06	2.4 (0.8–5.7)	0.059	2	0.13	14.9 (1.8–53.7)	0.008
		All other deaths	448	440.15	1.0 (0.9–1.1)	0.361	38	31.58	1.2 (0.9–1.7)	0.146
30–49	Male	Lung cancer	52	6.31	8.2 (6.2–10.8)	< 0.001	13	1.60	8.1 (4.3–13.9)	< 0.001
		Bronchiectasis	3	0.25	12.0 (2.4–34.9)	0.002	2	0.05	36.4 (4.1–132)	0.001
		Other COPD	4	1.52	2.6 (0.7–6.7)	0.068	2	0.56	3.6 (0.4–12.9)	0.108
		All other deaths	399	448.15	0.9 (0.8–1.0)	0.991	326	324.07	1.0 (0.9–1.1)	0.465
30–49	Female	Lung cancer	16	3.38	4.7 (2.7–7.7)	< 0.001	3	1.03	2.9 (0.6–8.5)	0.087
		Bronchiectasis	1	0.07	13.9 (0.2–77.1)	0.070	7	0.14	50.1 (20.0–103)	< 0.001
		Other COPD	4	0.90	4.4 (1.2–11.3)	0.014	5	0.37	13.7 (4.4–32)	< 0.001
		All other deaths	252	212.73	1.2 (1.0–1.3)	0.005	162	125.64	1.3 (1.1–1.5)	0.001
30–49	Pooled	Lung cancer	68	9.69	7.0 (5.4–8.9)	< 0.001	16	2.63	6.1 (3.5–9.9)	< 0.001
		Bronchiectasis	4	0.32	12.4 (3.3–31.7)	< 0.001	9	0.19	46.2 (21.1–87.7)	< 0.001
		Other COPD	8	2.42	3.3 (1.4–6.5)	0.004	7	0.92	7.6 (3–15.6)	< 0.001
		All other deaths	651	660.88	1.0 (0.9–1.1)	0.655	488	449.71	1.1 (1.0–1.2)	0.039

Abbreviations: E, expected; O, observed.

**Table 2:** Smoking habits among men and women in the two major cities in region II in 1990 compared with data for the rest of Chile [no. (%)].

		Smoking habits (cigarettes/day)					
	Nonsmokers	Occasional	1–9	10–19	≥ 20	Unknown	Total
Antofagasta	163,500 (76.4)	13,223 (6.2)	27,445 (12.8)	7,845 (3.7)	1,800 (0.8)	270 (0.1)	214,083 (100)
Calama	92,214 (80.4)	8,268 (7.2)	10,944 (9.5)	1,788 (1.6)	1,233 (1.1)	222 (0.2)	114,669 (100)
Rest of Chile	5,443,466 (75.1)	581,686 (8.0)	837,878 (11.6)	228,617 (3.2)	109,421 (1.5)	46,215 (0.6)	7,247,283 (100)

Data were obtained from the Ministerio de Planificación y Coordinación Nacional Republica de Chile IIIa, Encuesta Caracterización Socio Económica Nacional (1992).
